# Management of Stridor in Severe Laryngomalacia: A Review Article

**DOI:** 10.7759/cureus.29585

**Published:** 2022-09-26

**Authors:** Dhriti Jain, Shraddha Jain

**Affiliations:** 1 Otolaryngology, Jawaharlal Nehru Medical College, Datta Meghe Institute of Medical Sciences, Wardha, IND; 2 Otorhinolaryngology, Jawaharlal Nehru Medical College, Datta Meghe Institute of Medical Sciences, Wardha, IND

**Keywords:** stridor, gastroesophageal, supraglottoplasty, management, laryngomalacia

## Abstract

Laryngomalacia is the term most broadly used to portray the "internal breakdown of structures of supraglottis of the larynx at the time of inspiration. It is often associated with stridor during inspiration, which is of a high pitch at the time of birth and comes into notice by 14 days. When there is an increase in breathing, stridor worsens, and it is usually position-dependent. Laryngomalacia means the weakening of the larynx resulting in a collapse of the laryngeal cartilages, especially the epiglottis, into the airway. This partially occludes the upper airway during inspiration and causes inspiratory stridor. The exact etiology of the condition is not known. It is a well-known cause of noisy breathing in neonates and infants. The common presentation is a neonate with flushing and high-pitched inspiratory stridor that is usually noticed before 14 days of age. This worsens with breathing and supine positioning and improves in a prone position. Less commonly, it can present with hypoxia, feeding problems, aspiration, and failure to thrive. The condition may increase in severity during early life but usually self-resolves by two years of age. The hiccup-like squeak of laryngomalacia during inspiration is due to unsettled air flowing through the laryngeal passage. The condition is diagnosed with laryngoscopy, and the treatment varies with presentation and severity. Neonates with the uncomplicated disease can be treated expectantly. Those presenting with feeding problems and gastroesophageal reflux will require acid suppression. Severe complications like aspiration, severe airway obstruction, and hypoxia will require surgical treatment, including supraglottoplasty. In cases where the surgical treatment failed, noninvasive ventilation can be advised. The article reviews the various medical and surgical interventions and the management of severe laryngomalacia.

## Introduction and background

Laryngomalacia is the weakening of the laryngeal structures above the glottis, especially the epiglottis. This results in the inward collapse of these structures into the upper airway during inspiration, partially obstructing the airway [1]. Stridor is the high-pitched squeaking sound produced when air passes through an obstructed upper airway. Laryngomalacia affects 40%-75% of neonates and is the most frequent cause of stridor in infants [2,3]. The child usually presents with the symptom of stridor during inspiration which subsides on its own in the first 14 days of life. The symptoms can exacerbate by lying on the back, distress, and feeding [4]. The infant can also regurgitate, gag reflex, and fail to succeed [5,6]. The structures above true vocal cords, the supraglottis, are estimated to happen optional to the remarkable embryologic improvement of the laryngeal framework. There are defects in the nervous system along with anatomy and cartilaginous defects of the larynx [7]. The symptoms appear at birth or the first few days of life, peak at 6-7 months if not resolved by that time, and subside by two years of age [2]. The diagnosis of laryngomalacia is made within 4-5 months after birth [8]. Neonates with laryngomalacia may struggle with sucking, swallowing, and breathing during feeding because of the obstruction in air passage [9]. The expanded metabolic interest of organizing eating and breathing against the impediment can be so severe that it results in weight reduction and an inability to flourish. The extreme conditions may include cor pulmonale, right heart strain, and pectus excavatum. Due to obstruction of respiratory passage, chronic hypoxia may result in pulmonary hypertension.

The high tone of stridor can be observed at the time of inspiration or expiration or both (biphasic). When there is airway blockage at or above vocal cords, it is inspiratory stridor. And when there is blockage of the airway below the level of vocal cords, it is biphasic stridor. Viral croup is the most well-known cause of biphasic stridor in infants. There are 70%-90% minor cases of laryngomalacia with isolated and irregular stridor with the absence of cough, difficulty breathing, etc. Stridor is caused by a partial blockage of the laryngotracheal airway and is characterized by a loud, grating sound.

The signs in severe cases which require surgical management are inadequate weight increase (likely the main factor), dyspnea accompanied by severe or persistent intercostal or xiphoid retraction and respiratory distress episodes, etc.

Laryngomalacia can range in severity from minor to severe, depending on how it affects one's ability to be fed and breathe [8]. Patients with mild disease and a baseline resting SAO2 of less than 96% are projected to move to the moderate disease category in addition to experiencing reflux-related symptoms [10]. The most apparent symptom of upper airway blockage is frequently stridor, a sound produced by irregular airflow during breathing. It is commonly heard during inspiration (probably due to supraglottic or glottic blockage). Still, it can also be heard during expiration (perhaps due to severe upper airway obstruction and obstruction at or below the glottic level). Congenital abnormalities may cause stridor from birth, or they may appear over days, weeks, or months. Different congenital and acquired illnesses are common in neonates, babies, kids, and teenagers, and they must be recognized. A preliminary diagnosis is frequently made using the patient's history, age, and physical examination. Flexible airway endoscopy is the preferred diagnostic method; however other investigations may be required to make a particular diagnosis.

## Review

Etiology

**Figure 1:**
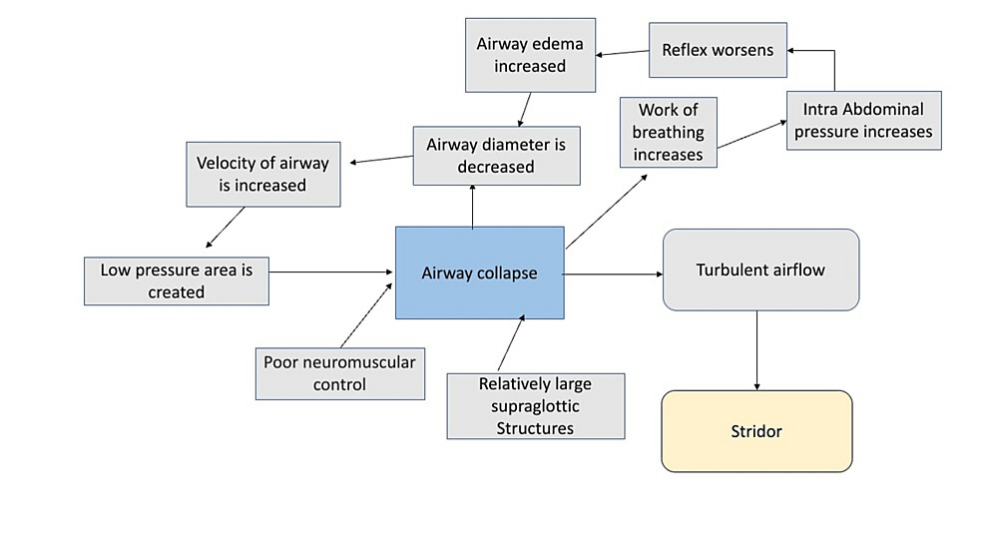
Mechanism of laryngomalacia

The larynx begins to form in the embryo at four weeks gestation and by eight weeks, all of the critical cartilage components are present [11]. Although the cause of the illness is not entirely understood, short aryepiglottic folds, a lengthy, curved epiglottis, and extra arytenoid mucosa prolapsing into the glottis are also present with anatomical anomalies usually linked to it [4]. Depending on where the abnormality is located, there are three different forms of laryngomalacia [4]. Type 1 results from superfluous arytenoid mucosa protruding into the glottis. Short aryepiglottic folds cause Type 2, and the posterior collapse of the epiglottis across the glottis causes Type 3 [12]. The specific cause of laryngomalacia is unknown, and this remains a topic of intense interest and investigation. The anatomic, cartilaginous, and neurologic theories of etiology are some examples. According to the anatomic explanation, stridor is caused by abnormally placed flaccid tissue. The difficulty with the anatomic explanation is that some newborns with laryngomalacia, a typical anatomic laryngeal finding, do not exhibit symptoms of airway blockage. According to the cartilaginous theory, the larynx's cartilages are young and unusually elastic. The discovery of histologically normal cartilage in neonates with symptomatic laryngomalacia has disproved this notion. According to the neurologic perspective, the peripheral nerves and brainstem nuclei in charge of breathing and airway patency, as well as their aberrant integration, may cause laryngomalacia. Laryngomalacia probably disappears as the infant grows older because the CNS system develops. The vagal nerve reflex, known as the laryngeal adductor reflex, controls laryngeal tone and function. The superior laryngeal nerve, located in the aryepiglottic fold, mediates the reflex's afferent activation [8]. The mechanism of laryngomalacia is shown in Figure 1. 

Diagnosis

The usual clinical history raises suspicion of laryngomalacia, but an awake newborn must undergo flexible laryngoscopy to confirm the diagnosis, even in the outpatient clinic. Flexible laryngoscopy may be performed with minimal assistance in the otolaryngology office. In either an upright or semi-reclined position, the baby is in the lap of the caregiver while a flexible laryngoscope is inserted via the nose and down to the larynx. Otolaryngologists can distinguish laryngomalacia from other causes of inspiratory stridor, such as vocal cord paralysis or a laryngeal cyst, by observing the dynamic movement of the laryngeal structures during spontaneous breathing. Laryngomalacia is characterized by blockage and supraglottic tissue collapse during inspiration [13]. In case of severe laryngomalacia where endoscopy is advised, hospitalization is recommended. Nasal endoscopy may be performed before general anesthesia induction for proper examination of the larynx of neonates under ideal circumstances (a sedated infant) and to identify any diseases that may be exacerbated by or related to sleep. To confirm the condition as laryngomalacia, direct laryngoscopy and bronchoscopy need to be performed.

Reassurance 

In most cases, laryngomalacia self-resolves by two years of age. For complete resolution, follow-up and reassurance are important. Medical and surgical treatments are considered in severe and persistent cases. If laryngomalacia is associated with some signs and symptoms, the treatment modality is chosen accordingly.

Medical management of gastroesophageal reflux

Between 65 and 100 percent of newborns with laryngomalacia have gastroesophageal reflux [11]. Due to the obstruction of the airway in laryngomalacia, there is a negative intrathoracic pressure that encourages gastric acid to reflux onto the tissues of the laryngopharynx, resulting in laryngopharyngeal reflux [8]. While the first approach holds that gastroesophageal reflux disease (GERD) causes laryngomalacia by disrupting the usual intrathoracic pressure gradient, which is often protective against reflux, the latter perspective holds that laryngomalacia causes GERD through increasing labour of breathing [4]. Various studies used esophageal biopsies, barium reflux on a barium swallow test, pH probes, and other methods to diagnose GERD. The various diseases were correlated with the existence of GERD in a patient, as shown in Table 1 [14].

**Table 1:** Diseases correlated with GERD The table shows diseases correlated with gastroesophageal reflux disease and their percentage of occurrence.

Diseases	Percentage
Subglottic stenosis	65
Supraglottic collapse	67
Posterior glottis edoema	70
Vocal fold edoema	88

Treatment options for gastroesophageal reflux disease include lifestyle modifications, medical treatment, and surgical management. Anti-reflux medication should be administered due to the frequency and aggravating role of pharyngolaryngeal reflux (PLR) in neonates with laryngomalacia. Thickened milk, maintaining posture after feedings, refraining from drinking before lying down, increasing the head of the bed or mattress, and antacids in infants who regurgitate must always be implemented as lifestyle and dietary measures [15].

Particularly thickening agents have been demonstrated to only reduce overt regurgitation, not the length of the esophageal pH being acidic [16]. For children above one year of age, there are no specific lifestyle changes, but factors that relieve the symptoms are considered [17]. Proton pump inhibitors (PPI), histamine-2 (H2) receptor blockers, and prokinetic medicines make up the majority of pharmacologic treatment [18]. Rapid tachyphylaxis (decrease in response per dosage), irritability, headaches, and head banging in certain newborns are side effects of long-term H2 blocker usage [19]. Proton pump inhibitors like omeprazole are also advised postoperatively for neonates who have undergone supraglottoplasty operations. Neonates who have gastro-oesophageal reflux have been documented to develop postoperative stenosis; however, the pathogenic nature of gastro-oesophageal reflux is only hypothesized at this point. H2 blockers are also associated with an increased risk of necrotizing enterocolitis in preterm newborns [20,21]. The cornerstone of reflux therapy for children is proton pump inhibitors. PPIs are superior to H2 blockers, as shown by several large-scale investigations, and their greater effectiveness is a result of their capacity to prevent meal-induced acid release [22]. PPIs like omeprazole are advised to treat GERD in infants in the hope that it will reduce the symptoms [23]. Also, even once a daily dose combined with lifestyle changes can occasionally result in sufficient acid suppression [24].

Supraglottoplasty

The most widely used surgical management for laryngomalacia to treat stridor is supraglottoplasty. An intravenous and mask anesthetic is used to put the patient to sleep. To rule out secondary lesions of the subglottis and trachea, rigid endoscopy is used to assess the airway in the first place. During spontaneous breathing, the supraglottis may be seen, and the main sites of collapse are recognized. The supraglottoplasty is then carried out with a focus on the excision of the extra arytenoid mucosa after the larynx has been exposed, utilizing operating laryngoscopes. This treatment has a low complication rate and approximate success rate of 94%. Technical advancements since the initial descriptions of supraglottoplasty, have mostly focused on the location and size of the tissues that need to be removed [2,25]. The technique which is under surgical management comprises of separation of short aryepiglottic folds, removal of extra supra-arytenoid mucosal tissue, division of the median glossoepiglottic ligament with the suspension of the epiglottis to the base of the tongue, partial epiglottectomy or combining any of the techniques [26,27]. Although supraglottoplasty is often performed bilaterally, several writers have discussed the benefits and drawbacks of unilateral vs. bilateral supraglottoplasty and have found a decreased risk of supraglottic stenosis following unilateral supraglottoplasty [28]. The various methods that can be used to respect excess tissues include cold micro instruments, CO2 laser, diode laser, coblation, etc. [26,29]. In certain fields, supraglottoplasty done with standard tools has positive outcomes, and the use of new tools is by trained individuals [30-32]. The conditions in which surgical treatment is required are failure to flourish, which is represented by the inability to gain weight with the markers on the growth chart. Neonates with this condition may not flourish because of difficulty in breathing, severe reflux, or improper feeding due to breathlessness [33]. The most common surgical treatment performed is the separation of aryepiglottic folds, along with the removal of extra aura arytenoid mucosa [34]. Carbon dioxide laser surgery is the use of carbon dioxide laser preferred by some surgeons for the treatment, despite its difficulty for anesthetists [35]. The most observed anomaly of the larynx is laryngomalacia which has a good prognosis [36]. The neonates who require surgical treatment are those presenting with severe laryngomalacia [37]. The symptoms along with stridor that is severe define the need for surgery. When laryngomalacia is associated with these symptoms, surgery is advised: Difficulty in breathing, respiratory agony, obstructive sleep apnoea, difficulty in feeding associated with suffocation, and inability to gain weight.

Noninvasive ventilation in laryngomalacia

This procedure has been considered useful to treat obstruction of the upper airway along with hypoxia in children and neonates with sleep apnea due to obstruction and chronic stridor because of laryngomalacia [38-40]. This procedure lowers the efforts for breathing and precise pressure support, along with positive end-expiratory pressure, and is considered effective [41]. Gaseous exchange is improved at night, and also the minimum required height and weight gain occur due to noninvasive ventilation. The need for tracheotomy and its consequences, like difficulty in developing speech, obstruction of cannula, distress, etc., can be avoided [42,43].

One-and-a-half coblation supraglottoplasty

This method is effective and minimally invasive in treating laryngomalacia with stridor. The aryepiglottic folds are often separated bilaterally to enlarge the supraglottic airway when type II is treated. Although uncommon, bilateral supraglottoplasty might result in supraglottic stenosis because of the existence of two raw surfaces in opposite directions [2]. As the name suggests, one-and-a-half coblation surglottoplasty indicates that one aryepiglottic fold is cut while causing scarring of the lateral side related to the other fold.

Tracheostomy

The severe cases of laryngomalacia, which are not managed by any other method, require tracheostomy. It is usually done to save the life of a critically ill child. Neonates who compromise the airway require this procedure [44]. Other situations which require tracheostomy in neonates are severe laryngomalacia, prolonged ventilation, trauma, stenosis of the laryngotracheal airway, etc. [45]. This procedure has many complications. The first several days following the surgery are crucial in tracheostomies. As the stoma or tract is not properly developed at this time, there is a chance that the tube will be inserted into a false tract rather than the trachea, making it harder to recannulate the tracheostomy tube after an accident. Subcutaneous emphysema, pneumothorax, and postoperative bleeding are some acute postoperative problems. Infants have these issues at a greater rate than older people. A horizontal skin incision 1.5 cm long and one fingerbreadth above the sternal notch is made with the patient in hyperextension. For improved visualization and to reduce the risk of secondary wound infection, subcutaneous fat is removed. Splitting the midline raphe is then used to gradually divide the strap muscles. The tracheal fascia is opened following identification [46].

## Conclusions

Laryngomalacia is a common condition in neonates which causes stridor. Usually, it appears two weeks after delivery. In order to diagnose a condition the larynx must be seen during breathing. Either primary or secondary type is possible. The standard of care is conservative, particularly in cases of secondary laryngomalacia. Just 10% of cases call for surgical intervention. Supraglottoplasty is quite successful and seldom causes postoperative complications. There are several management options for severe laryngomalacia, which include conservative management, medical treatment with proton pump inhibitors, etc., to treat gastroesophageal reflux, and surgical management like surglottoplasty, and carbon dioxide laser surgery. Surgery controls symptoms associated with laryngomalacia. A non-invasive ventilation procedure is done when a patient fails to respond to surgical management. Tracheostomy is done in very severe cases of laryngomalacia.

## References

[1] Fattah HA, Gaafar AH, Mandour ZM (2011). Laryngomalacia: Diagnosis and management. ejenta.

[2] Richter GT, Thompson DM (2008). The surgical management of laryngomalacia. Otolaryngol Clin North Am.

[3] Holinger LD (1980). Etiology of stridor in the neonate, infant and child. Ann Otol Rhinol Laryngol.

[4] Olney DR, Greinwald JH Jr, Smith RJ, Bauman NM (1999). Laryngomalacia and its treatment. Laryngoscope.

[5] Srikanthan A, Scott S, Desai V, Reichert L (2022). Neonatal airway abnormalities. Children (Basel).

[6] Leonard JA, Reilly BK (2022). Laryngomalacia in the premature neonate. Neoreviews.

[7] Munson PD, Saad AG, El-Jamal SM, Dai Y, Bower CM, Richter GT (2011). Submucosal nerve hypertrophy in congenital laryngomalacia. Laryngoscope.

[8] Thompson DM (2007). Abnormal sensorimotor integrative function of the larynx in congenital laryngomalacia: a new theory of etiology. Laryngoscope.

[9] Richter GT, Wootten CT, Rutter MJ, Thompson DM (2009). Impact of supraglottoplasty on aspiration in severe laryngomalacia. Ann Otol Rhinol Laryngol.

[10] Thompson Thompson, Dana M (2010). Laryngomalacia: factors that influence disease severity. Curr Opin Otolaryngol Head Neck Surg.

[11] Baxter MR (1994). Congenital laryngomalacia. Can J Anaesth.

[12] Friedman EM, Vastola AP, McGill TJ, Healy GB (1990). Chronic pediatric stridor: etiology and outcome. Laryngoscope.

[13] Mancuso RF, Choi SS, Zalzal GH, Grundfast KM (1996). Laryngomalacia. The search for the second lesion. Arch Otolaryngol Head Neck Surg.

[14] May JG, Shah P, Lemonnier L, Bhatti G, Koscica J, Coticchia JM (2011). Systematic review of endoscopic airway findings in children with gastroesophageal reflux disease. Ann Otol Rhinol Laryngol.

[15] Haver K, Brigger M, Hardy S (2009). Pediatric aerodigestive disorders. https://books.google.co.in/books?hl=en&lr=&id=_Fo0BwAAQBAJ&oi=fnd&pg=PR5&dq=Pediatric+Aerodigestive+Disorders.&ots=BClsygDmPE&sig=30mn7yJx7N7PiqwWo51vOoArhTM#v=onepage&q=Pediatric%20Aerodigestive%20Disorders.&f=false.

[16] Khoshoo V, Ross G, Brown S (2000). Smaller volume, thickened formulas in the management of gastroesophageal reflux in thriving infants. J Pediatr Gastroenterol Nutr.

[17] Vandenplas Y, Rudolph CD, Di Lorenzo C (2009). Pediatric gastroesophageal reflux clinical practice guidelines: joint recommendations of the North American Society for Pediatric Gastroenterology, Hepatology, and Nutrition (NASPGHAN) and the European Society for Pediatric Gastroenterology, Hepatology, and Nutrition (ESPGHAN). J Pediatr Gastroenterol Nutr.

[18] Preston C, Donnellan C, Moayyedi P (2001). Medical treatments for the short term management of reflux oesophagitis. Cochrane Database Syst Rev.

[19] Orenstein SR, Shalaby TM, Devandry SN (2003). Famotidine for infant gastro-oesophageal reflux: a multi-centre, randomized, placebo-controlled, withdrawal trial. Aliment Pharmacol Ther.

[20] Guillet R, Stoll BJ, Cotten CM, Gantz M, McDonald S, Poole WK, Phelps DL (2022). Association of H2-blocker therapy and higher incidence of necrotizing enterocolitis in very low birth weight infants. Pediatrics.

[21] Denoyelle F, Mondain M, Gresillon N, Roger G, Chaudre F, Garabedian EN (2003). Failures and complications of supraglottoplasty in children. Arch Otolaryngol Head Neck Surg.

[22] Kahrilas PJ, Shaheen NJ, Vaezi MF (2008). American Gastroenterological Association Medical Position Statement on the management of gastroesophageal reflux disease. Gastroenterology.

[23] (2022). The Royal Children's Hospital Melbourne: Gastrooesophageal reflux disease in infants. https://www.rch.org.au/clinicalguide/guideline_index/Gastrooesophageal_reflux_disease_in_infants/.

[24] Reichel O, Keller J, Rasp G, Hagedorn H, Berghaus A (2022). Efficacy of once-daily esomeprazole treatment in patients with laryngopharyngeal reflux evaluated by 24-hour pH monitoring. Otolaryngol Head Neck Surg.

[25] Lane RW, Weider DJ, Steinem C, Marin-Padilla M (1984). Laryngomalacia. A review and case report of surgical treatment with resolution of pectus excavatum. Arch Otolaryngol.

[26] Zalzal GH, Collins WO (2005). Microdebrider-assisted supraglottoplasty. Int J Pediatr Otorhinolaryngol.

[27] Golz A, Goldenberg D, Westerman ST, Catalfumo FJ, Netzer A, Westerman LM, Joachims HZ (2000). Laser partial epiglottidectomy as a treatment for obstructive sleep apnea and laryngomalacia. Ann Otol Rhinol Laryngol.

[28] Reddy DK, Matt BH (2001). Unilateral vs. bilateral supraglottoplasty for severe laryngomalacia in children. Arch Otolaryngol Head Neck Surg.

[29] Ayari-Khalfallah S, Fuchsmann C, Froehlich P (2008). Thulium laser in airway diseases in children. Curr Opin Otolaryngol Head Neck Surg.

[30] Holinger LD, Konior RJ (1989). Surgical management of severe laryngomalacia. Laryngoscope.

[31] Jani P, Koltai P, Ochi JW, Bailey CM (1991). Surgical treatment of laryngomalacia. J Laryngol Otol.

[32] Polonovski JM, Contencin P, Francois M, Viala P, Narcy P (1990). Aryepiglottic fold excision for the treatment of severe laryngomalacia. Ann Otol Rhinol Laryngol.

[33] Toynton SC, Saunders MW, Bailey CM (2001). Aryepiglottoplasty for laryngomalacia: 100 consecutive cases. J Laryngol Otol.

[34] Fajdiga I, Beden AB, Krivec U, Iglic C (2008). Epiglottic suture for treatment of laryngomalacia. Int J Pediatr Otorhinolaryngol.

[35] Rita L, Seleny F, Holinger LD (1983). Anesthetic management and gas scavenging for laser surgery of infant subglottic stenosis. Anesthesiology.

[36] Fearon B, Ellis D (1971). The management of long term airway problems in infants and children. Ann Otol Rhinol Laryngol.

[37] Roger G, Denoyelle F, Triglia JM, Garabedian EN (1995). Severe laryngomalacia: surgical indications and results in 115 patients. Laryngoscope.

[38] Guilleminault C, Pelayo R, Clerk A (1995). Home nasal continuous positive airway pressure in infants with sleep-disordered breathing. J Pediatr.

[39] Waters KA, Everett FM, Bruderer JW, Sullivan CE (1995). Obstructive sleep apnea: the use of nasal CPAP in 80 children. Am J Respir Crit Care Med.

[40] Fauroux B, Pigeot J, Polkey MI, Roger G, Boulé M, Clément A, Lofaso F (2001). Chronic stridor caused by laryngomalacia in children: work of breathing and effects of noninvasive ventilatory assistance. Am J Respir Crit Care Med.

[41] Rapoport DM (1996). Methods to stabilize the upper airway using positive pressure. Sleep.

[42] Dubey SP, Garap JP (1999). Paediatric tracheostomy: an analysis of 40 cases. J Laryngol Otol.

[43] Wetmore RF, Marsh RR, Thompson ME, Tom LW (1999). Pediatric tracheostomy: a changing procedure?. Ann Otol Rhinol Laryngol.

[44] Ozmen S, Ozmen OA, Unal OF (2009). Pediatric tracheotomies: a 37-year experience in 282 children. Int J Pediatr Otorhinolaryngol.

[45] Itamoto CH, Lima BT, Sato J, Fujita RR (2010). Indications and complications of tracheostomy in children. Braz J Otorhinolaryngol.

[46] Corbett HJ, Mann KS, Mitra I, Jesudason EC, Losty PD, Clarke RW (2007). Tracheostomy--a 10-year experience from a UK pediatric surgical center. J Pediatr Surg.

